# Decision Support App for Managing Behavioral and Psychological Symptoms of Dementia in Nursing Homes

**DOI:** 10.1093/geroni/igaf122.2520

**Published:** 2025-12-31

**Authors:** EunKyo Kim, Eunhee Cho, Minhee Yang, Sinwoo Hwang, Jungwon Cho, SangA Lee

**Affiliations:** Yonsei University, Seoul, Republic of Korea; Yonsei University, Seoul, Republic of Korea; Yonsei University, Seoul, Republic of Korea; Yonsei University, Seoul, Republic of Korea; Yonsei University, Seoul, Republic of Korea; Yonsei University, Seoul, Republic of Korea

## Abstract

Managing behavioral and psychological symptoms of dementia (BPSD) is an increasing global challenge. This study aimed to test an app developed for managing BPSD in a nursing home setting. A cluster randomized controlled trial included 35 older adults with dementia and 28 nurses across 11 facilities. Over eight weeks, the intervention group used an app that recorded and predicted BPSD. The app analyzed BPSD patterns using machine learning and provided individualized non-pharmacological interventions such as music, reminiscence, and exercise therapy. However, the control group used the BPSD recording system without access to individualized interventions. Overall BPSD, depression, agitation, and nurses’ BPSD management competencies were measured. Nurses in the intervention group recorded their narrative experiences using the app, as well as residents’ reactions to it. Generalized mixed models revealed reductions in depression and agitation scores over time for both groups without a group-by-time interaction. Overall BPSD decreased in the control group, showing a group-by-time interaction. Nurses in both groups showed improvement in BPSD management competencies. Based on their experience using the app, nurses considered playing residents’ preferred songs during music therapy to be helpful in maintaining focus and emotional stability. Additionally, reminiscence therapy supported cognitive engagement by connecting past experiences to the present through photographs. While the app had a user-friendly interface, technical constraints—such as slow processing and limited content—reduced its effectiveness. Future research should focus on improving technical performance, diversifying content, and adapting the app for varied care settings such as adult daycare centers.

